# Autoimmune Thrombocytopenia Treated by Low‐Dose Splenic Irradiation Followed by Splenectomy in a Patient With Systemic Lupus Erythematosus

**DOI:** 10.1002/ccr3.71661

**Published:** 2025-12-08

**Authors:** Ryosuke Hanaoka

**Affiliations:** ^1^ Department of Rheumatology Kamitsuga General Hospital Kanuma Tochigi Japan

**Keywords:** autoimmune thrombocytopenia, refractory ITP, splenectomy, splenic irradiation, systemic lupus erythematosus

## Abstract

Severe autoimmune thrombocytopenia (ATP) in systemic lupus erythematosus (SLE) patients can be life‐threatening when refractory to standard treatments. Low‐dose splenic irradiation (LDSI) can provide temporary platelet recovery before definitive splenectomy. A 26‐year‐old woman with newly diagnosed SLE presented with severe thrombocytopenia (12,000/μL) and life‐threatening bleeding. Despite multiple therapies, including prednisolone, cyclophosphamide, cyclosporine A, danazol, rituximab, and high‐dose IVIg, thrombocytopenia persisted. LDSI (15 Gy in 15 fractions over 5 weeks) was performed, leading to rapid platelet recovery and enabling safe splenectomy, resulting in long‐term remission. LDSI can serve as an effective bridging therapy in refractory ATP associated with SLE, allowing safer splenectomy and sustained remission.

## Key Clinical Message

Low‐dose splenic irradiation followed by splenectomy can be an effective treatment option for refractory autoimmune thrombocytopenia in patients with systemic lupus erythematosus when standard therapies fail.

## 1 Introduction

Systemic lupus erythematosus (SLE) is a chronic autoimmune disease of unknown etiology that can affect virtually any organ system. Hematologic manifestations, including leukopenia, autoimmune hemolytic anemia, and thrombocytopenia, are common among the diverse organ disorders observed in individuals with SLE. The most frequent cause of severe thrombocytopenia in SLE is autoimmune thrombocytopenia (ATP), characterized by immune‐mediated destruction of platelets and megakaryocytes.

Patients with thrombocytopenia typically present with mucocutaneous bleeding manifestations, including petechiae, purpura, ecchymoses, epistaxis, gingival bleeding, and menorrhagia. In cases of severe thrombocytopenia, more serious bleeding complications, such as gastrointestinal hemorrhage, hematuria, or intracranial bleeding, may occur. While mild thrombocytopenia (platelet counts 100,000‐150,000/μL) occurs in 25%–50% of SLE patients, severe thrombocytopenia (platelet counts < 10,000/μL) with major bleeding is rare but requires urgent multidisciplinary management.

Here, we report a case of refractory severe ATP in an SLE patient who experienced life‐threatening bleeding episodes, where low‐dose splenic irradiation was successfully employed as bridging therapy to enable subsequent definitive splenectomy.

## 2 Patient Information

A 26‐year‐old Japanese woman was admitted to our hospital for the evaluation of severe thrombocytopenia. She had no significant past medical history except for being born prematurely at 29 weeks of gestation, requiring surgery for patent ductus arteriosus and retinopathy of prematurity. Her family history was notable for Sjögren syndrome in her mother. She had no known allergies and was taking no regular medications prior to admission.

## 3 Clinical Findings

The patient complained of general malaise persisting for several months before admission, without other obvious clinical manifestations of SLE. Ten days prior to admission, she sustained a fall with transient loss of consciousness, resulting in a scalp injury. Emergency evaluation revealed pancytopenia despite red blood cell and platelet transfusions and granulocyte‐colony stimulating factor administration. Given a positive antinuclear antibody, she was referred to our hospital for further investigation.

On physical examination, she was 157 cm tall, weighed 40 kg, with a temperature of 36.6°C, a pulse rate of 80/min, and a blood pressure of 110/50 mmHg. A large subcutaneous hematoma was observed on her right forehead from the recent fall. Palpebral conjunctiva appeared anemic. No oral mucosal ulceration was present. Her spleen was enlarged 15 mm below the left costal margin.

Laboratory investigations revealed: hematocrit 29.1% (reference 34.8–45.0), hemoglobin 8.2 g/dL (11.5–15.0), platelet count 12,000/μL (140,000‐340,000), and white blood cell count 2500/μL (3300‐9000) with differential showing 2.0% bands, 72.0% neutrophils, 17.0% lymphocytes, 6.0% monocytes, 0.0% eosinophils, 1.0% basophils, and 2.0% atypical lymphocytes. Antinuclear antibody demonstrated a speckled pattern with titers exceeding 1:2560. Additional positive findings included rheumatoid factor (77 U/mL), anti‐Smith antibody (22.8 U/mL), anti‐U1‐ribonucleoprotein antibody (146 U/mL), platelet‐associated immunoglobulin G, and urea breath test for *Helicobacter pylori* . Antiphospholipid antibody testing showed negative results for anticardiolipin IgG, anti–β2‐glycoprotein I–dependent anticardiolipin IgG, and lupus anticoagulant by the diluted Russell's viper venom time method. Cytomegalovirus (CMV) IgG was positive, indicating past infection; hepatitis B surface antigen, surface antibody, and core antibody were all negative; hepatitis C antibody was negative.

## 4 Diagnostic Assessment

Abdominal computed tomography and ultrasonography confirmed splenomegaly without splenic calcification, focal lesion, or signs of portal hypertension. Bone marrow examination revealed increased immature megakaryocytes with strong cytoplasmic basicity, with minimal platelet adhesion observed. No dysplastic changes were identified. Chromosome analysis by G‐banding showed no chromosomal abnormalities. Based on clinical presentation, laboratory findings, and bone marrow characteristics, we diagnosed SLE with secondary ATP.

## 5 Therapeutic Intervention

Initial treatment included oral prednisolone 40 mg daily and intravenous cyclophosphamide 500 mg. With institutional ethics committee approval for off‐label use, we additionally administered cyclosporine A 200 mg daily, danazol 400 mg daily, and four weekly courses of rituximab 500 mg. These medications, while supported by academic literature, lacked insurance coverage for SLE treatment in Japan at that time. *Helicobacter pylori* eradication was also performed following the positive breath test.

Despite these interventions, cytopenia showed insufficient improvement. High‐dose intravenous immunoglobulin (IVIg) was administered expecting temporary platelet count elevation to enable safe splenectomy; however, adequate improvement for surgical intervention did not occur. The patient progressively developed joint pain, subcutaneous bleeding in the lower extremities, and significant irregular genital bleeding, requiring repeated emergency platelet transfusions.

Given the refractory nature of thrombocytopenia and ongoing bleeding complications, we obtained ethics committee approval for off‐label use of low‐dose splenic irradiation (LDSI). The patient received 15 Gy delivered in 15 fractions over 5 weeks (three fractions weekly).

## 6 Follow‐Up and Outcomes

Platelet count began increasing immediately following LDSI initiation and recovered to normal range upon treatment completion. Platelet counts remained normal for an extended period without requiring immediate splenectomy. We recommended splenectomy during this stable period, but the patient initially declined consent.

Several months later, when the platelet count began declining again, she consented to splenectomy, which was performed successfully with a platelet count of 80,000/μL. The procedure was completed using a combination of a laparoscopic approach and a small laparotomy without significant adhesion‐related complications from prior irradiation.

**FIGURE 1:**
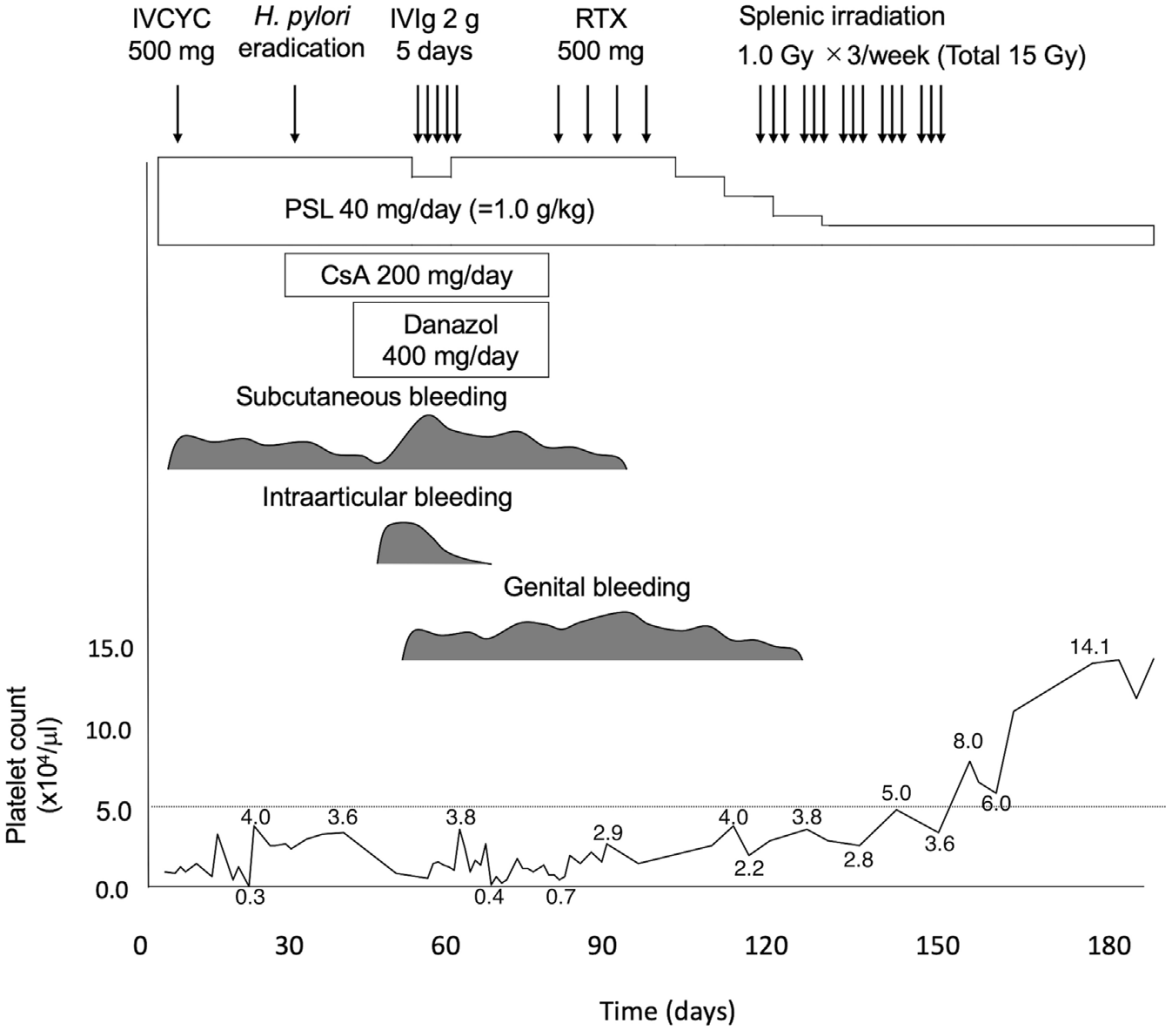
Clinical course showing platelet count response to various treatments. Timeline demonstrates refractory thrombocytopenia despite multiple therapies (prednisolone [PSL], intravenous cyclophosphamide [IVCYC], cyclosporine A [CsA], rituximab [RTX], high‐dose intravenous immunoglobulin [IVIg]), followed by successful response to low‐dose splenic irradiation and sustained remission after splenectomy. Platelet counts (×10^3^/μL) are indicated along the curve to illustrate temporal changes during treatment.

Following splenectomy, her platelet count stabilized within the normal range, and she has remained in clinical remission during subsequent follow‐up. The detailed clinical course, including platelet recovery and treatment response, is illustrated in Figure 1.

## 7 Discussion

The most commonly employed agents for initial ATP treatment are glucocorticoids and IVIg. Second‐line treatments include splenectomy, rituximab, thrombopoietin receptor agonists (TPO‐RAs), or immunosuppressive therapy for patients with continued clinically significant bleeding, platelet counts < 10,000–20,000/μL, or selected patients with counts 20,000–30,000/μL after initial treatment [1].

In our case, LDSI was performed prior to splenectomy because persistent severe thrombocytopenia prevented safe surgical intervention despite multiple treatment approaches. TPO‐RAs were not utilized as this case occurred before these agents received insurance approval for this indication in Japan, although they were available in other countries.

LDSI exerts both immunomodulatory and cytoreductive effects. The proposed mechanisms include induction of lymphocyte apoptosis within the splenic parenchyma, modulation of autoreactive B‐ and T‐cell populations, suppression of splenic macrophage activity, and reduction in platelet sequestration within the spleen [2, 3]. These combined effects can lead to transient amelioration of autoimmune platelet destruction and facilitate a safer window for surgical intervention. The dose used in this case (15 Gy in 15 fractions) was within the commonly reported range associated with immunomodulatory rather than myelosuppressive effects.

LDSI represents an alternative to splenectomy, similar to partial splenic embolization, primarily described in elderly patients at high surgical risk, with effectiveness reported in approximately two‐thirds of cases [2, 3, 4, 5, 6, 7, 8, 9, 10, 11, 12]. Several case reports have described LDSI for ATP in HIV‐positive patients, but few reports exist for SLE patients.

A theoretical concern with LDSI involves postirradiation adhesion development that might complicate subsequent splenectomy. However, our patient underwent splenectomy without significant adhesion‐related complications. LDSI's relative safety stems from the radiation tolerance of adjacent structures such as the colon and left kidney being well above doses used in this treatment.

Recent years have witnessed a shift toward treatments with reduced immunosuppression, such as TPO‐RAs, a trend accelerated by the COVID‐19 pandemic [1]. Nevertheless, refractory cases persist, highlighting the importance of individualized treatment approaches based on patient age, platelet count, bleeding risk, and comorbidities.

Although LDSI is less commonly utilized in difficult‐to‐treat ATP with SLE given the availability of rituximab or TPO‐RAs, refractory cases still exist. Moreover, LDSI can be safely incorporated as a component of reduced‐intensity conditioning regimens for hematopoietic stem cell transplantation in myelofibrosis [13]. LDSI may be considered a useful treatment preceding safe splenectomy in ATP patients with SLE when thrombocytopenia remains insufficiently improved with other treatments.

At the time of this case, hydroxychloroquine was not covered by Japanese national insurance for SLE treatment, precluding its use. While additional intravenous cyclophosphamide courses are commonly considered in refractory SLE, we were concerned that cumulative bone marrow suppression might exacerbate the patient's already critical thrombocytopenia, leading us to avoid repeated administration.

Similarly, while mycophenolate mofetil is widely used internationally for SLE, it was only approved for posttransplant immunosuppression in Japan during this patient's treatment period and was inaccessible for autoimmune indications. Consequently, cyclosporine A and danazol were selected as alternative immunosuppressive agents based on existing efficacy evidence in refractory autoimmune thrombocytopenia and after ethics committee approval for off‐label use.

One potential limitation of our interpretation involves the timing of rituximab administration, which concluded approximately 30 days prior to LDSI initiation. Rituximab's maximal therapeutic effect typically occurs 4–8 weeks after infusion, suggesting a possible overlapping or synergistic effect. Therefore, while the temporal association strongly suggests LDSI was the primary driver of the platelet response, a contributory role for rituximab cannot be ruled out. The two therapies may have acted synergistically, with rituximab suppressing autoreactive B cells and LDSI further reducing splenic immune activation and platelet sequestration.

## 8 Conclusion

This case demonstrates that LDSI can serve as an effective bridging therapy for refractory ATP in SLE patients when standard treatments fail. The technique enabled safe subsequent splenectomy and achieved sustained clinical remission. While newer agents like TPO‐RAs have expanded treatment options, LDSI remains a valuable consideration for selected patients with refractory disease, particularly when surgical intervention is planned but contraindicated due to severe thrombocytopenia.

## Author Contributions

**Ryosuke Hanaoka:** writing – original draft, writing – review and editing.

## Funding

The author has nothing to report.

## Consent

Written informed consent was obtained from the patient for publication of this case report and any accompanying images.

## Conflicts of Interest

The author declares no conflicts of interest.

## Data Availability

No data are available due to patient confidentiality.
